# Digital epidemiology of high-frequency search listening trends for the surveillance of subjective well-being during COVID-19 pandemic

**DOI:** 10.3389/fpsyg.2024.1442303

**Published:** 2024-11-01

**Authors:** Khalida Al-Kenane, Frederic Boy, Ahmad Alsaber, Rania Nafea, Shihanah AlMutairi

**Affiliations:** ^1^Business School, Gulf University for Science and Technology, Mubarak Al-Abdullah, Kuwait; ^2^Zienkiewicz Institute for AI, Data & Modelling & NAIADES Research Network School of Management, Swansea University, Swansea, United Kingdom; ^3^College of Business and Economics, American University of Kuwait (AUK), Salmiya, Kuwait; ^4^Seneca Polytechnic, School of Human Resources and Global Business, Toronto, ON, Canada

**Keywords:** digital epidemiology, lockdown, anxiety, psychological distress, global shock, Kuwait

## Abstract

**Background:**

The coronavirus disease (COVID-19) pandemic has led to a dramatic increase in online searches related to psychological distress. Governments worldwide have responded with various measures to mitigate the impact of the virus, influencing public behavior and emotional well-being. This study investigated the relationship between government actions and public reactions in terms of online search behaviors, particularly concerning psychological distress during the pandemic. The primary objective of this study was to analyze how changes in government policies during the COVID-19 pandemic influenced public expressions of psychological distress, as reflected in the volume of related online searches in Kuwait.

**Method:**

Utilizing Google Trends data, the study analyzed search frequencies for terms associated with psychological distress such as “anxiety” and “lockdown.” The analysis correlated these search trends with government actions using the Oxford COVID-19 Government Response Tracker (OxCGRT). The study period covered March 1, 2020, to October 10, 2020, and involved extensive data collection and analysis using custom software in R programming.

**Results:**

There was a significant correlation between the stringency of government-imposed restrictions and the volume of online searches related to psychological distress. Increased searches for “lockdown” coincided with heightened government restrictions and were associated with increased searches for “anxiety,” suggesting that policy measures significantly impacted public psychological distress.

**Conclusion:**

The study concludes that governmental responses to the COVID-19 pandemic, measured through OxCGRT, have a measurable impact on public psychological distress, as evidenced by online search behaviors. This underscores the importance of considering psychological impacts in policymaking and suggests further research to explore this dynamic comprehensively. Future studies should focus on refining the correlation between specific types of policy measures and different expressions of psychological distress to better inform public health strategies and interventions.

## Introduction

In today’s interconnected world, search engines have become an essential part of daily life, with millions of users conducting queries across a vast range of topics to seek information. When examined closely, these search behaviors can provide valuable insights into societal trends, potentially reflecting broader economic conditions and quality of life. Traditional macroeconomic indicators, such as Gross Domestic Product (GDP), offer a snapshot of a nation’s economic health but often fail to capture the intricacies of human well-being (Diener et al., 2010). For nearly a century, it has been recognized that while GDP measures market-based transactions, it neglects critical factors like leisure time, income inequality, and access to healthcare—key elements that contribute to overall societal well-being (Brodeur et al., 2021).

As a result, there has been growing demand for more comprehensive metrics that better account for the complexities of human flourishing. However, developing reliable and timely well-being measures remains a challenge, as many existing metrics depend on census surveys, which are often outdated and prone to human bias. To address these limitations, researchers are increasingly turning to digital epidemiology, utilizing real-time data from search engines to analyze societal trends. By studying the temporal patterns of keyword searches, researchers aim to create more accurate and timely indicators of societal well-being (Diener et al., 2010).

In this regard, Google Trends has emerged as a valuable tool, offering a normalized index of search traffic for specific keywords across various geographic regions. This data has already been employed to track a range of phenomena, from public health concerns during the COVID-19 pandemic to changes in mental well-being. Researchers are now investigating whether keyword search volumes can be used to predict self-reported well-being, providing a new approach to monitoring societal trends in near real-time. Trends data offers a real-time indicator of public interest in topics related to psychological distress. Although search terms like “anxiety” or “depression” do not directly reflect an individual’s emotional state, they capture public concern or curiosity about these issues. Even if some searches are out of curiosity, spikes in search volume often correspond with real-world stressors, making them a useful tool for tracking collective psychological responses. For instance, during the COVID-19 pandemic, increases in anxiety-related searches often coincided with rising cases, lockdowns, or other stressors, offering insights into societal stress levels. Following the unprecedented global COVID-19 pandemic, societies worldwide have faced numerous challenges, leading to diverse government responses aimed at mitigating the virus’s impact (Bell et al., 2015; Orben and Przybylski, 2019). As nations grappled with the multifaceted repercussions of the pandemic, individuals sought solace and information, often turning to online platforms to express and explore their concerns (Bell et al., 2015; Orben and Przybylski, 2019). This research endeavors to illuminate the intricate relationship between societal psychological distress, as manifested in weekly-averaged search listening time-series for the keyword “Anxiety” in both English and Arabic, and the dynamic actions of governments in response to the pandemic.

Central to our investigation was the utilization of the Oxford Covid-19 Government Response Tracker (OxCGRT), a comprehensive tool developed by the University of Oxford (Hale et al., 2021). This tracker systematically captures and evaluates government interventions across 20 indicators, encompassing containment and closure policies, economic measures, and health-system responses. Ranging from school closures to vaccination policies, the OxCGRT provides a nuanced perspective on the multifaceted strategies adopted by governments worldwide (Hale et al., 2021).

The novel aspect of this research lies in its pioneering attempt to bridge the realms of digital behavior and government actions, offering a unique insight into how societal expressions of anxiety, as evidenced through online search trends, temporally align with the evolving landscape of governmental responses to the pandemic. By establishing a correlation between search-listening patterns and government actions, this study aims to contribute substantially to our understanding of the interplay between individual psychological distress and broader societal dynamics during a global crisis. Additionally, a study investigating the effects of physical activity (PA) levels and dietary habits on sleep quality among elite and amateur athletes highlights the broader implications of maintaining physical health during the pandemic. It found that while elite athletes maintained higher PA levels, amateurs had better dietary habits, and a sense of control over the pandemic significantly influenced sleep quality, further emphasizing the importance of holistic well-being during crises. By establishing a correlation between search-listening patterns and government actions, this study aims to contribute substantially to our understanding of the interplay between individual psychological distress and broader societal dynamics during a global crisis (Taheri et al., 2023b). Furthermore, Taheri et al. (2023a) investigate how the pandemic impacted the mental and physical health of elite athletes in Iran. The study revealed that during the lockdown, athletes experienced heightened anxiety and stress, alongside moderate depression levels. Additionally, there was a reduction in physical activity, and many participants, particularly those with lower activity levels, exhibited emotional eating behaviors. The results indicate that engaging in high-intensity physical activity can help alleviate some of the adverse psychological effects of the pandemic, highlighting the need for strategies aimed at enhancing the mental and physical well-being of elite athletes during such difficult times (Taheri et al., 2023a).

As we embark on this unprecedented exploration, it is our hope that the findings will not only shed light on the intricate connections between online expressions of anxiety and governmental interventions, but also pave the way for future investigations into the psychological dimensions of public responses to crises.

## Methodology

This study used Google Trends data to analyze the temporal patterns of searching for information related to emotional distress. It is important to acknowledge that, in all current research utilizing Google Relative Search Volume (RSV) data, researchers must explicitly demonstrate that Google Inc. has supplied time-series data. However, the methodology used to determine the relative frequencies of searches has not been fully disclosed owing to commercial confidentiality constraints.

Furthermore, it is crucial to exercise careful discretion in selecting suitable variables when examining the correlation between Google Trends data and fluctuations in online manifestation of psychological distress. Anxiety and depression are among the most traditional markers of psychological distress, and are commonly recurring themes in public discourse, despite not being clinical or technical terms. Hence, the specific language queries chosen for data mining in this study encompassed ‘anxiety,’ ‘depression,’ ‘divorce,’ ‘stress,’ and ‘suicide.’

The chosen keywords were evaluated and translated by the researcher, and then checked by a professional language expert in Kuwait (Al-Diwan Translation Center, Hawally, Bin Khaldoun Street, Al-Ghareeb Complex, Office 1, Kuwait City).

It is essential to acknowledge that the persistent global disturbances brought about by the Covid-19 pandemic have led to an ongoing change in human behavior, largely driven by the adverse effects of the pandemic on individuals’ emotional well-being. Consequently, specific keywords related to the pandemic and its related occurrences were integrated during the data-collection phase. Furthermore, it is noteworthy that certain terms used for data retrieval displayed regional discrepancies in Kuwait. Thus, to accommodate these variations, dual synonyms were assigned to certain words instead of relying on a single translation, recognizing that different social groups may employ distinct terminology, as depicted in Table 1.

**Table 1:** English and Arabic words used for search listening.

English keyword	Arabic translation	Synonym	Arabizi
Anxiety	القلق	التوتر	Al8ala8
Stress	ضغط		Daght
Divorce	طلاق	انفصال	Altala8
Depression	كآبة		Ka2aba
Suicide	انتحار		Ante7ar
Anger	غضب		Ghadab
Sadness	حزن		7azin
Coronavirus	فيروس كورونا		---
Death	الموت		Almawt
Unemployment	البطالة		Alba6ala
Happiness	السعادة		Alsa3ada
World ending	نهاية العالم		Nihayat al3alam
Starvation	مجاعة		Maja3a
When will the coronavirus end?	متى ينتهي فيروس كورونا		-----
Pandemic	وباء		Waba’
Quarantine	الحجر الصحي		Al7ajer alse7i
Loneliness	الوحدة		Alwa7da
Insomnia	الأرق		Alara8
Judgment day	يوم القيامة		Youm Al8eyama
Coronavirus vaccine	لقاح فيروس كورونا		----
Second wave	الموجة الثانية		Almawja althanya
Lockdown	Not found in Arabic		
Breakdown	Not found in Arabic		
Recession	Not found in Arabic		

### Control keywords

**Figure 1:**
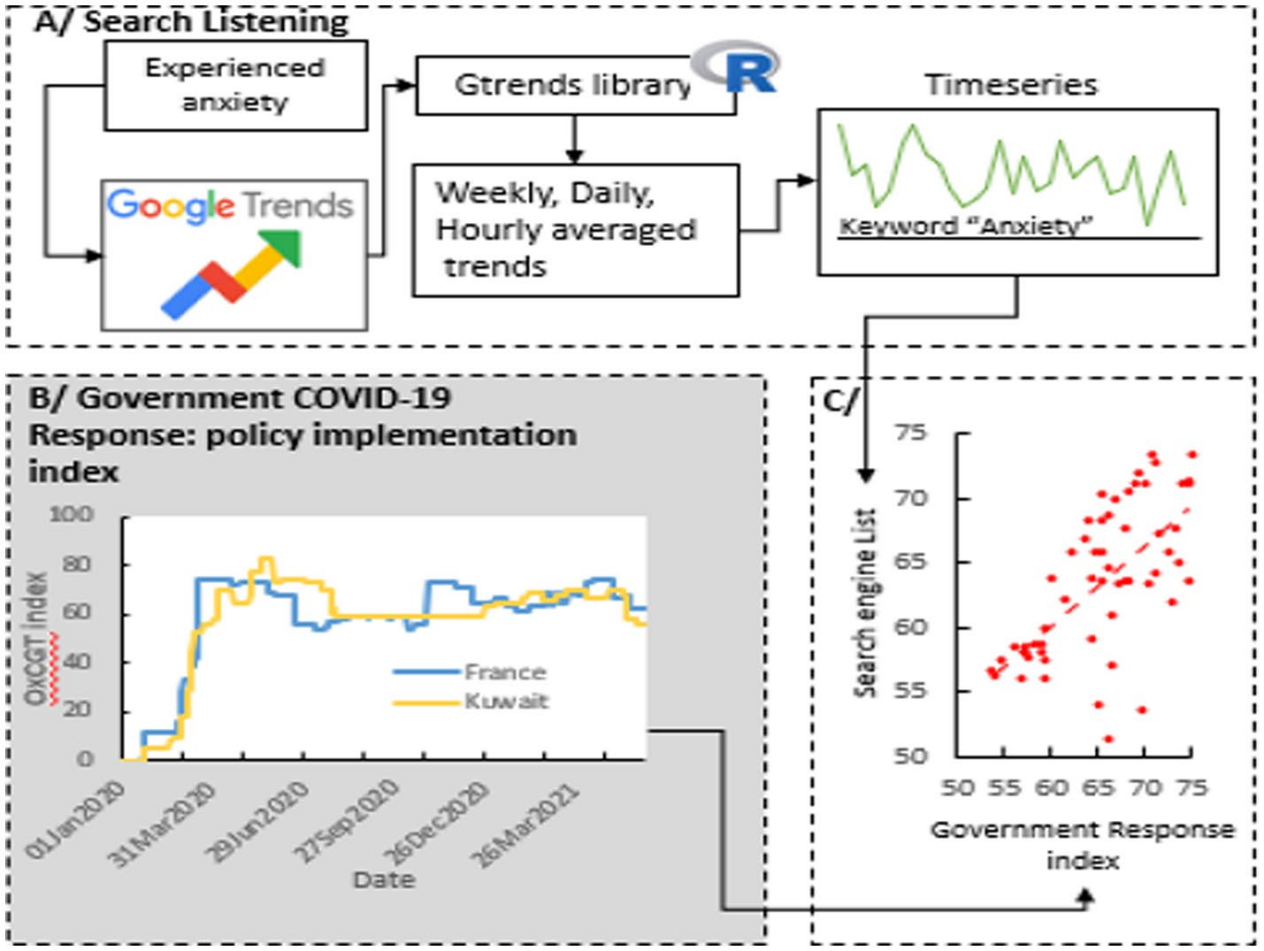
Overview of the SUPER-API data architecture: surveillance of personal experience for the real time appraisal of policy implementation.

To ensure the credibility of the conclusions and insights drawn from analyzing the collected search data, it is crucial to demonstrate that searching for neutral terms would produce a clear pattern, or possibly none at all. If random words show no recognizable pattern, this indicates that the insights derived from analyzing the search data are significant and can be used to create predictive models. This validation step was conducted as part of the project. It is worth noting that the frequently collected time-series data often show overlaid seasonal patterns. Hourly data typically exhibit three types of regular occurrence: daily, weekly, and seasonal. However, forecasting models face challenges with daily and weekly patterns, as they typically follow an annual cycle with a seasonal period averaging approximately 52.179 weeks. Furthermore, the Weekly Opinions and Lifestyle Survey assessed the percentage of adults reporting significant anxiety in a representative sample of the British population from May 21, 2020, to December 13, 2020. Consequently, the researchers evaluated Google Trends (Relative Search Volume) for several emotionally charged keywords. The findings from combining these two datasets in the analysis are presented later in this paper (Figure 1).

Furthermore, it is vital to visually illustrate the temporal changes in the expression of online psychological distress during hypothesis formulation. This helps to ensure that searches for neutral terms display clear temporal patterns. The words chosen as controls for this study are as follows (Table 2).

**Table 2:** Control neutral keywords used for the study.

Control keywords
Orange
Red
Green

## Results

Using Google Trends for search analysis provides valuable data on general trends in human behavior and specific patterns related to global events, serving as a useful tool for decision-making.

**Figure 2:**
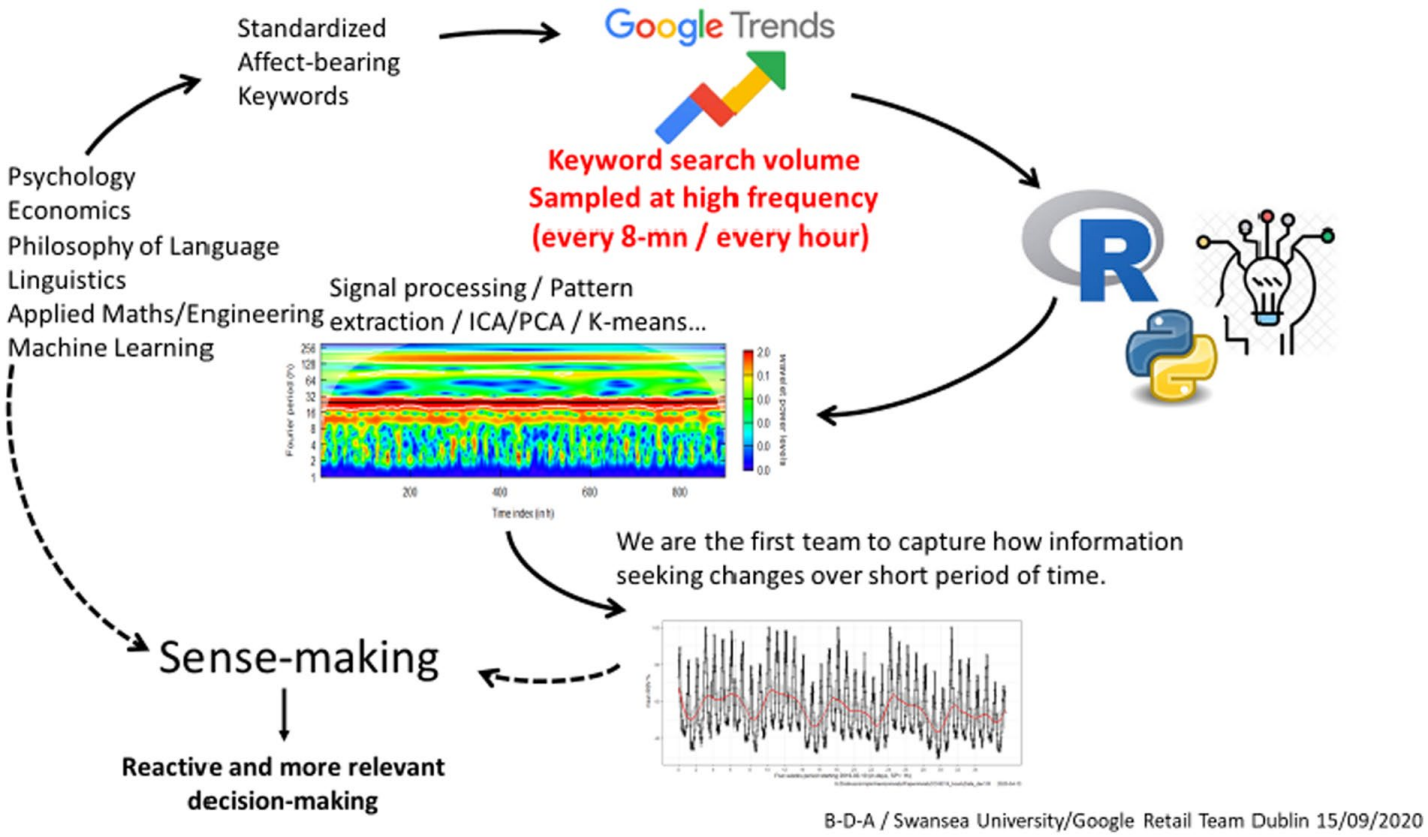
General framework for collecting and analyzing search listening data.

**Figure 3:**
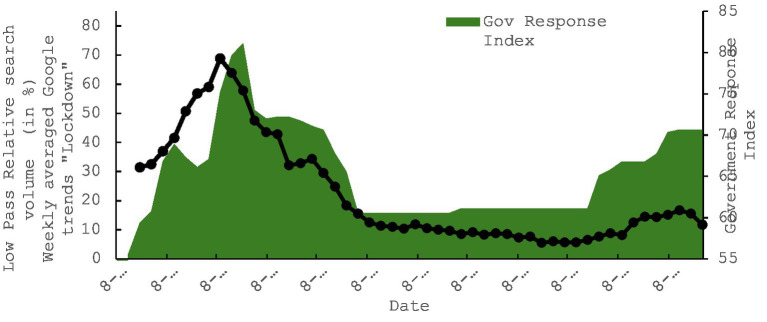
Co-evolution of the timeseries for the keyword “Lockdown” and the government response index in Kuwait (OxCGRT) between the weeks starting 08/03/2020 and the 28/02/2021.

**Figure 4:**
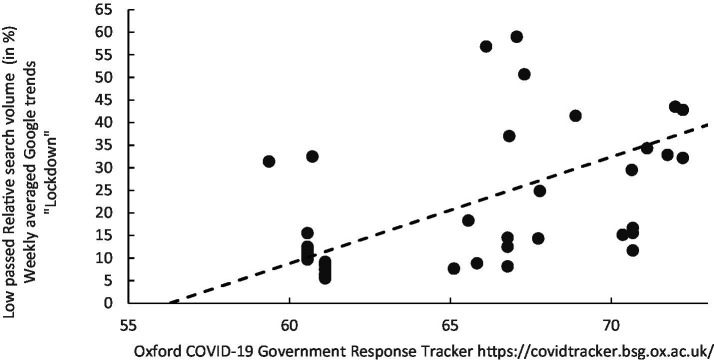
Scatterplot of the weekly relative search volume for the keyword “Lockdown” against the COVID-19 Government-Response Tracker (OxCGRT) for Kuwait (weekly measurement between 08/03/2020 and 28/02/2021).

When discussing the factors influencing psychological well-being during the pandemic, it is appropriate to cite the work of Diotaiuti et al. (2023) to provide additional context on the importance of cognitive appraisals and perceived self-efficacy. The approach to search listening analysis adopted in this research begins with the identification of accurate and standardized keywords associated with the target trait being studied, which, in this case, is subjective well-being. This identification process involves considering various aspects such as psychology and economics, typically through a review of the literature and an understanding of events occurring in different geographic regions of interest. It is also advantageous to include synonyms when conducting search listening tasks. Once identified, Google Trends was used to gather the frequency of search data for each keyword. The collected data can then be analyzed for correlations and patterns following the process detailed in Figures 2–4.

The present research aims to reveal how weekly-averaged search listening time-series for the keyword “Anxiety” (taken as the average for the keyword “anxiety” in the English language, and its Arabic translation) correlates (time-locked) to the actions of the government as computed by the COVID-19 Government-Response Tracker (OxCGRT).^1^ The latter tool is useful, as governments have adopted a wide range of measures in response to the COVID-19 outbreak. This tool was designed to track and compare policy responses on a global scale consistently and rigorously. Known as the Oxford COVID-19 Government Response Tracker (OxCGRT), it gathers publicly available information on 20 indicators concerning government actions. These indicators cover various aspects, including containment and closure policies (e.g., school closures and movement restrictions), economic policies (e.g., income support and foreign aid), and health system policies (e.g., COVID-19 testing protocols, healthcare investments, and vaccination strategies). These data play a crucial role in understanding the correlation between online expressions of psychological distress and societal changes. To the best of our knowledge, the research presented in this paper is original and pioneering, aimed at providing initial validation for our analysis of search trends.

### Exploration of the relationship between search listening and the Oxford University COVID-19 Government-Response Tracker (OxCGRT) index in Kuwait

#### Search listening for “lockdown” and OxCGRT

**Figure 5:**
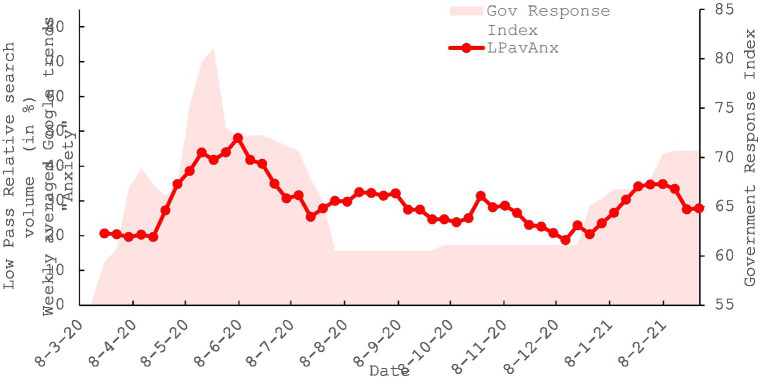
Co-evolution of the time-series for the keywords “anxiety” in English and Arabic (Average of the search volume for the keyword Anxiety in English and Arabic) and the OxCGRT government response index calculated for Kuwait between the weeks starting 08/03/2020 and the 28/02/2021.

**Figure 6:**
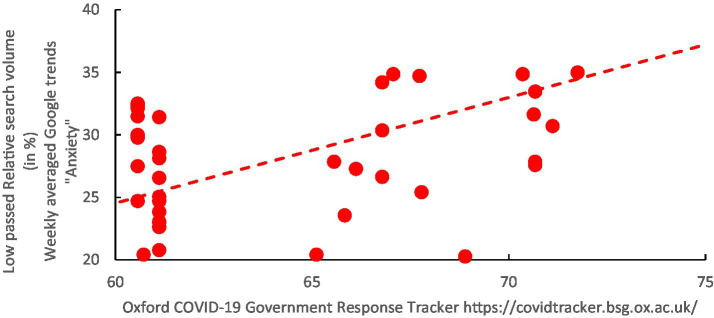
Scatterplot of the weekly-averaged relative search volume for the average keywords “Anxiety” in English and Arabic against the COVID-19 Government-Response Tracker (OxCGRT) calculated for Kuwait (weekly measurement between 08/03/2020 and the 28/02/2021).

Initially, we established a reasonable baseline by examining the relationship between Kuwaiti searches for the keyword ‘lockdown’ and the OxCGRT index. The parallel evolution of these two factors is evident when visually observing their progression (Figures 4–6). The co-evolution profile can be quantified, revealing a strong correlation with a Pearson’s R value of 0.7143 (with a bias of −0.0026 and a standard error of 0.078), and a *p*-value less than 0.00001. This correlation suggests that the prominence of the keyword’ lockdown” in an individual’s online searches aligns with significant government communication and intervention on a large scale. Additionally, we conducted non-parametric bootstrap analyses with 1,000 replications to assess the robustness of this correlation. The resulting 95th percentile confidence intervals, {0.5422, 0.8433}, did not encompass a value of zero, indicating that this condition was met, and the correlation was considered robust, even in the presence of deviations from normality. This preliminary analysis enables us to examine how web searches related to the prominent contextual issue of lockdown implementation directly correlate with government actions (Table 3).

**Table 3:** COVID-19 restrictions in Kuwait and policy dates between 08/05/2020 and 08/02/2021.

Date	Event or governmental response
February 24, 2020	First COVID-19 cases announced in Kuwait.
March 11, 2020	Kuwait started a voluntary stay at home approach suspending all work across government sectors except emergency services.
March 14, 2020	Commercial air travel was suspended.
March 22, 2020	Partial curfew was implemented: hours were between 5 pm-4 am.
April 6, 2020	Curfew was amended from 4 am-6 pm.
April 24, 2020	The start of the holy month of Ramadan. The partial curfew was further amended from 4 pm–8 am.
May 8, 2020	On this day, it was announced that starting from May 10, 2020, the country would be under a full curfew, meaning that nobody would be permitted to leave their homes at all.
July 15, 2020	The land borders were opened.
July 17, 2020	Mosques were reopened.
August 1st, 2020	Airports were fully reopened and operational
August 18, 2020	Kuwait transitioned into the fourth phase of its five-phase plan to resume normal activities. During this phase, salons, gyms, barbershops, tailors, and spas reopened, and restaurants were allowed to expand their services.
August 30th, 2020	The Kuwaiti government lifted a nationwide partial curfew, while activities including celebrations, parties, weddings, gatherings, banquets, and funerals were to remain restricted to curb the spread of the coronavirus.
September 8th, 2020	Kuwait reported 857 new coronavirus cases in the last 24 h, marking a significant increase compared to the summer months and causing concern among the population. Social media platforms were abuzz with discussions suggesting the possibility of implementing a curfew if the number of cases continues to rise.
October 8th, 2020	With cases in Kuwait rising daily, news reports indicated that if the daily case count reached 1,000, airports would be closed again, and additional restrictions would be put in place. This news brought worry to citizens who had recently emerged from a prolonged lockdown period.
November 8th, 2020	November was a relatively calm month in terms of news, with few events causing stress or anxiety. It was a period of relative relaxation for many.
December 8th, 2020	In early December, Kuwait was experiencing favorable numbers with no significant rise in cases or a high number of deaths. However, between December 20th and January 4th, Kuwait’s airport abruptly closed again without prior notice due to the rapid spread of the new variant originating from the UK. This development sparked fear in Kuwait, especially since many students had recently flown back from the UK for the Christmas break.
January 8th, 2021	At the start of the month, Kuwait faced no significant issues until a ban list was issued, prohibiting flights from 34 countries, including the UK. Additionally, there were discussions of potentially imposing a curfew in response to the prevailing situation.
February 8th, 2021	As cases rapidly surged, Kuwait took decisive measures, including halting the entry of non-Kuwaitis into the country. By mid-month, restaurants ceased offering dine-in services, transitioning to takeaway only. Subsequently, hairdressers, barbershops, and gyms were shut once again. On March 7th, 2021, a curfew was imposed from 5 pm to 5 am as part of efforts to curb the spread of the virus.

The table outlines the sequence of events that unfolded in Kuwait from March 8, 2020, to February 28, 2021. These events likely influenced the relative search volumes owing to the implementation of stringent guidelines. The OXCGRT index serves as a metric to quantify this information, reflecting governmental responses and their impact on various aspects of society and public behavior.

#### Search listening for “anxiety” and OxCGRT

Once a baseline has been established, it becomes possible to examine the frequency of searches for the keyword “anxiety,” considering both the English and Arabic versions of the term. These searches are known to correlate with the expression of psychological distress on social media and are synchronized with the OxCGRT index. Interestingly, a strong and statistically significant correlation was found, with a Pearson’s R value of 0.64810 (bias = −0.007, SE = 0.085) and a *p*-value less than 0.00001. Additionally, the 95th percentile non-parametric confidence intervals were calculated to be {0.4375, 0.7797}.

#### Search listening for the keyword’s “anxiety” and “lockdown” in Kuwait

**Figure 7:**
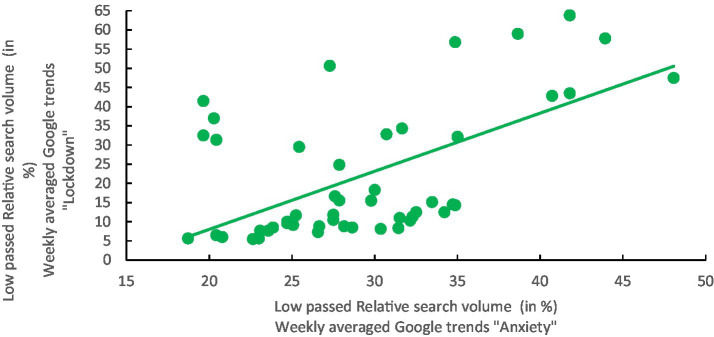
Scatterplot for the weekly relative search volume for the average keywords “Anxiety” in English and Arabic against the weekly relative search volume for the keyword “Lockdown” for Kuwait (weekly measurement between 08/03/2020 and 28/02/2021).

To complete the current analysis, we explored how searches for the keywords “Lockdown” and “Anxiety” covaried in Kuwait during the same period (Figures 4–7). A Pearson’s correlation of 0.4782 (bias = −0.008, SE = 0.13), with a *p*-value of less than 0.0004, was found, indicating that the saliencies of both keywords were temporally co-evolving, as expected. Furthermore, non-parametric bootstrap analyses (with 1,000 replications) were conducted to assess the robustness of the detected correlations. The resulting 95th percentile confidence intervals, {0.2209, 0.7516}, did not encompass a zero value, indicating robustness, even in the presence of deviations from normality.

This preliminary analysis allowed us to examine how web searches related to psychological distress varied in conjunction with searches for a general information background (within the broader context of the COVID-19 pandemic). This underscores the idea that searching for information about psychological distress, as exemplified by searches for the keyword “Anxiety,” reflects the significance of an issue (“Lockdown”) to some extent in the general background information, as well as the actions of public authorities.

## Discussion

The COVID-19 pandemic has led to a significant surge in the utilization of digital technology (De’ et al., 2020) and intention to adopt new technologies (Al Reshaid et al., 2024) as individuals are subjected to stringent social distancing regulations and lockdowns. When using Google Trends data to analyze search behavior during events like the COVID-19 pandemic, it is important to acknowledge that this data is influenced by many external factors, such as media coverage, public awareness campaigns, or socio-economic conditions. However, there are several reasons to focus on Google Trends despite the presence of these confounding factors; Google Trends provides valuable real-time or near real-time data, which allows for an in-depth analysis of how people react to global events like COVID-19. During the pandemic, search behavior often reflected the public’s immediate concerns, such as anxiety about the virus or the demand for information on symptoms (Kornellia and Syakurah, 2023). This ability to observe shifts in public sentiment in real-time offers crucial insights, enabling timely interventions, although factors like media influence may also shape the response. With its large-scale data coverage, Google Trends collects data from vast numbers of users across different regions and demographics, making it a comprehensive tool for capturing broad societal behavior. While media and socio-economic factors may influence search behavior, the data includes responses from diverse groups, helping to balance biases introduced by local factors (Kornellia and Syakurah, 2023).

By analyzing patterns in search data, it’s possible to link spikes in specific searches to broader public concerns. For example, searches related to anxiety may align more closely with peaks in COVID-19 cases or new government regulations than with media coverage alone. Although the media plays a role in amplifying these issues, Google Trends offers insight into the organic public reaction to real-world conditions (Vaidyanathan et al., 2022). Furthermore, real-world events, such as lockdowns, surges in COVID-19 cases, or changes in government policy, often directly affect search behavior. Spikes in searches for terms like “anxiety” or “mental health” frequently correlate with these events, despite the media’s amplification. Google Trends effectively captures how the public responds to the real-world impact of such events, offering a reliable tool for tracking collective sentiment (Vaidyanathan et al., 2022).

The OxCGRT index for Kuwait indicates that when the government implements stricter restrictions across various sectors, including health and sports, there is a corresponding increase in searches for the term “lockdown’.”

The onset of the pandemic’s peak occurred around April 4, 2020, marked by the enforcement of unprecedented measures, such as evacuations to repatriate all students and citizens from abroad before airports were completely shut down. Additionally, during this period, the first full lockdown was imposed, resembling martial law, which was a novel experience for the younger generations. Finally, martial law was imposed in Kuwait during the Gulf War in 1990.

In December 2020, there wasn’t a significant peak in the search volume for “lockdown.” This decrease in the government index can be attributed to the discovery of a new variant. Several factors might have contributed to this trend. First, a lockdown may not have been imposed during that period, or it is possible that people began to adapt to the situation and became accustomed to the measures in place.

Additionally, many individuals have lost their jobs during this time. As previous studies have shown, unemployment has a detrimental effect on well-being, primarily due to loss of income. The influence of income loss on an individual’s well-being has been well-documented in the literature. This study supports previous findings indicating that job loss has significant adverse effects on well-being, with income loss being one of the primary causes (Abdel-Khalek and Korayem, 2018).

Indeed, research has consistently highlighted a robust connection between employment status and individuals’ levels of life satisfaction and happiness. Employment is frequently associated with higher levels of life satisfaction, while unemployment can significantly diminish life satisfaction (Binder and Coad, 2012; Boyce et al., 2010; Sharafeddine et al., 2024). These events underscore the profound impact on individuals’ well-being during challenging times, prompting them to turn to search engines to seek understanding and support for their feelings and experiences at those exact moments.

Additionally, it was intriguing to explore the correlation between the search volumes for the keywords “anxiety” and “lockdown.” The findings revealed a parallel relationship between the search volume for “lockdown” and “anxiety.” As previously mentioned, there was a direct correlation between the search for “anxiety” and psychological distress. Similarly, the data indicated a parallel correlation between the search volume for “lockdown” and “anxiety,” suggesting that the volume of searches for “lockdown” also correlates with psychological distress. This discovery aligns with previous studies that observed how placing individuals under restrictions influences their web searches when seeking assistance for psychological distress (Glowacz and Schmits, 2020; Diab-Bahman and Al-Enzi, 2020; Handique and Teronpi, 2022; Lindner et al., 2022; Ruotolo et al., 2023).

Evidence from this study suggests that the severity of government restrictions imposed on socializing and movement correlates with online indicators of psychological distress. However, it is important to interpret this correlation cautiously, especially regarding its directionality. Unanswered questions need to be investigated in the future.

One possible interpretation is that governments act in response to a perceived, predicted, or intended pre-emptive rise in online expressions of psychological distress among the population. Alternatively, it could be that the increase in online expressions of psychological distress stems from the public witnessing governments implementing emergency changes through media and official communication. Currently, it is difficult to draw definitive conclusions. However, this underscores the importance of investigating the future of governmental communication and its implications for the psychological well-being of the population. The novel framework introduced in this study proposes innovative approaches to address these critical questions and offers potential avenues for further exploration and understanding.

With a clear comprehension of the potential and constraints of correlational research, Research 4 endeavored to attain deeper insights and explore more nuanced details. This is done by examining the online expression of psychological distress in a context devoid of external circumstances that could influence an individual’s mental state. By isolating the online expression of psychological distress from external factors, Research 4 aims to uncover fundamental insights into how psychological distress manifests online, independent of external influences. This approach allows for a more granular examination of the underlying mechanisms and dynamics at play in online expressions of distress. By acknowledging both the potential and limitations of correlational research, Study 4 presents an alternative approach focused on delving into the intricacies of psychological distress expression in a controlled setting, thereby offering a deeper understanding of its fundamental nature in the online realm.

After analyzing search listening data in response to external circumstances triggered by the COVID-19 pandemic in Kuwait, it became intriguing to delve deeper into time-series data obtained at a higher frequency within the United Kingdom. This shift in focus allows for a more detailed exploration of how online behaviors and expressions of distress evolve over shorter time intervals in different geographic contexts.

The analysis of search listening data revealed interesting patterns, particularly regarding peaks occurring around midnight on Sunday. This timing may serve as a strong social marker, signaling the end of the weekend and the impending start of the workweek. Many individuals use Sunday nights to catch up on chores, errands, and preparations the week ahead, which can contribute to feelings of stress and anxiety. To alleviate this, it has been suggested that people spread their tasks throughout the week, allowing for more relaxation and enjoyment on Sundays. By adopting this approach, individuals can better prioritize self-care and social activities, which can positively impact mental health and overall well-being (Søvold et al., 2021).

Analyzing search listening data in response to external circumstances triggered by the COVID-19 pandemic in Kuwait has led to a shift in focus toward exploring time-series data at a higher frequency within the United Kingdom. This shift allows for a more detailed examination of how online behaviors and expressions of distress evolve over shorter time intervals in different geographic contexts. The analysis of search listening data uncovered intriguing patterns, particularly highlighting peaks around midnight on Sunday. This timing serves as a significant social marker, signaling the transition from weekends to workweeks. Many individuals utilize Sunday nights to catch up on tasks and prepare for the upcoming week, leading to heightened stress and anxiety. To address this, it is recommended that individuals distribute their tasks throughout the week, allowing for more relaxation and enjoyment on Sunday. By adopting this approach, individuals can prioritize self-care and social activities, positively impacting their mental health and overall wellbeing. Vahle-Hinz et al. (2014) emphasized the significant relationship between work stress at the end of a workday, work-related rumination in the evening, and subsequent restful sleep. Taylor et al. (2008) further supported these findings by indicating that delaying bedtime and wake-up time can result in a delayed circadian rhythm and decreased mood and cognitive functioning. Moreover, Borges et al. (2017) highlighted the association between night eating syndromes and emotional states, including depression, anxiety, and stress symptoms. Cousin et al. (2023) discussed the negative impact of night-shift work on mental health, which resonates with the potential distress observed in the context of online behaviors during late-night hours. Additionally, Pereira and Gross (2015) underscored how social stressors at work can impact sleep and recovery, potentially influencing the observed patterns on Sunday nights. In conclusion, the analysis of search listening data in the United Kingdom revealed intriguing patterns around Sunday nights, reflecting heightened stress and anxiety levels. These findings align with the existing research on work stress, rumination, sleep quality, and mental health outcomes. By spreading out tasks throughout the week and prioritizing self-care and relaxation on Sundays, individuals can mitigate the impact of stress and enhance their overall well-being (Hayes et al., 2022).

On Sundays, the lowest search volume occurs between 5 am and 8 pm, possibly because people tend to sleep after staying out late the night before. Once awake, they may focus on completing tasks, keeping them occupied and away from the online platforms. However, for the rest of the week, the highest search peak was recorded between 3 am and 5 am, indicating that many individuals were awake during these hours, possibly due to insomnia or overthinking life issues. During busy workday hours (6 am to 8 pm), search activity decreases as people become occupied with their job responsibilities.

After 5 pm, there is a rush to return home and have dinner, after which individuals may resume using their phones. As the evening progresses and tasks are completed, search activities increase again, possibly as people unwind before bed or seek solutions to stress and pressure related to work and daily life. Overall, these patterns highlight the complex interplay between daily routines, stressors, and online behavior, underscoring the importance of balancing and incorporating self-care practices into daily life.

Research on psychological distress among Arab populations has highlighted the impact of stressors, such as the COVID-19 pandemic, social integration challenges, and mental health stigma (Eshel et al., 2022; Lindheimer et al., 2020; Saravanan et al., 2020). Studies have shown that Arab individuals may somatize psychological distress and express emotional issues through their physical symptoms (Al-Krenawi, 2013; Hasan and Tumah, 2018). The prevalence of psychological distress in the Arab region is comparable to the global rate (Hamdan, 2009). The shift to online platforms has influenced the expression of distress, with social media environments impacting users’ emotional disclosures (Xu et al., 2023). Online education has been associated with increased anxiety and psychological distress among university students (Diab-Bahman and Al-Enzi, 2021; Mostafa et al., 2023; Al-Enzi et al., 2023). The COVID-19 pandemic has exacerbated psychological distress, affecting sleep quality and mental well-being during quarantine (Al-Rasheed et al., 2021). Culturally appropriate interventions are crucial in addressing psychological distress among Arab populations, considering the unique sociocultural adversities they face (Ahmed et al., 2011; AlBuloushi et al., 2024). Understanding the nuances of distress within the Arab context is essential for providing effective mental health support (Hassan et al., 2016; Lindheimer et al., 2020). Social support, both online and offline, plays a significant role in modulating distress levels, particularly among healthcare workers and patients (Fino et al., 2021). In conclusion, the temporal organization of online expressions of psychological distress in the Arab Peninsula is shaped by cultural, social, and environmental factors. By understanding these nuances, tailored interventions can be developed to support mental wellbeing in the Arab region.

Investigating how these differences influence individuals’ online expressions of psychological distress provides valuable insights into cross-cultural variations in coping mechanisms, stressors, and societal norms. By comparing these findings with those from the Western world, we can gain a deeper understanding of the universal and culturally specific aspects of psychological distress and its online expression. Moreover, exploring the temporal patterns in the Arab Peninsula allows the identification of unique features and trends that may inform tailored interventions and support systems to address mental health needs in this region. By acknowledging and embracing cultural diversity, we can develop more effective strategies to promote mental well-being and resilience in diverse populations.

### Data mining Kuwait

#### Stress and ضغط (translation: “stress; Arabizi “daght”)

The analysis reveals that the hours with the highest peak for the keyword “ضغط” (translation: “stress”; Arabizi “daght”) are between 12 am and 2:30 am. Subsequently, there was a significant drop in the search volume starting straight after 2 am and continuing until 6:30 am. Following this, the search volume remains relatively average until 6 pm, before beginning to escalate again. The data illustrate that the highest Relative Search Volume (RSV) was 52%, while the lowest was 37%. These patterns highlight fluctuations in online searches related to stress throughout the day, with notable peaks occurring during the late night and early morning hours.

The data presented in this study reveal a significant disparity in the search volume scale between the English and Arabic languages. The highest Relative Search Volume (RSV) observed in Arabic was 52%, which was notably higher than the highest RSV in English, which was only 35%. Furthermore, the lowest RSV in Arabic surpassed the highest RSV in English. The percentage difference between the RSV of the two highest points is 32%, indicating that the highest number of searches for “stress” occurs in Arabic in Kuwait. Given this discrepancy, the focus will be on the results obtained in Arabic.

The analysis revealed that the highest peak for the keyword “stress” occurs between 4 am and 7 am, with 5 am being the peak hour, and there is no sharp drop in search volume at any time for “stress.” The highest Relative Search Volume (RSV) was 35%, whereas the lowest RSV was 25%. These timings indicate a notable peak that may be considered abnormal in Western countries, and differences in patterns are apparent in the perceived data. This pattern is attributed to religious practices, particularly the call for morning prayer that wakes up most individuals in Kuwait. After awakening, individuals experiencing mental health conditions such as insomnia, depression, or anxiety may struggle to fall back asleep. Consequently, searching for such terms may serve as an escape or an attempt to find solutions. Moreover, in many Arabic countries, a lower social status is associated with higher religiosity. Individuals who wake up for morning prayer but end up using their phones may belong to this group and could experience depression or stress, particularly if they are unemployed or facing financial difficulties (Al-Kandari and Crews, 2014).

Further analysis suggests that nighttime searches may be associated with individuals’ economic status from a non-religious perspective. In Kuwait, English proficiency is predominantly linked to middle to upper classes, as it is primarily taught in private schools. Therefore, it is plausible to assume that individuals who conduct searches in English are either native speakers or speakers of other languages who hold higher-paying jobs and have prestigious positions that require them to retire early. Conversely, individuals conducting searches in Arabic during late hours may not have jobs that necessitate an early start, or may be dissatisfied with their current employment situation, leading to insomnia or other mental health conditions. This interpretation is supported by the fact that exceptional English skills are typically required to secure prestigious jobs in Kuwait, as English is extensively used in business and diplomatic spheres (Al-Rubaie, 2023; Dashti, 2015).

The highest peak observed between 12 am and 2 am can be attributed to significant lifestyle differences between Middle Eastern and Western countries, particularly in Kuwait. In Kuwaiti culture, it is common for the majority of people to not retire early to bed, even if they have an early start the next day. Many individuals may find themselves lying awake in bed at midnight, grappling with insomnia or depression, prompting them to turn on their phones and engage in online searches to occupy their minds. The sharp drop in searches between 2 am and 6:30 am suggests a need for rest, as individuals prepare to wake up early for work. In Kuwait, the workday typically begins at 7:30 am, earlier than that in Western countries, and ends at 2 pm in the public sector and 3:30 pm in the private sector. From 6:30 am to 6 pm, individuals are occupied with work, social engagements, and various activities, diverting their attention away from online searches related to stress or mental health issues.

After work, people typically return home for lunch and may take a nap to compensate for their lack of sleep during the night. In the evening, individuals engage in social activities, spend time with friends and family, or run errands until late hours. During these late night hours, when individuals are alone and the distractions of the day have subsided, searching for stress-related terms may occur again. Overall, the observed patterns reflect the cyclical nature of daily life in Kuwait, with peaks in online searches for stress-related terms occurring during late night hours when individuals have time to reflect and seek information or support for their mental health concerns.

The findings suggest that Kuwaitis may not perceive “stress” as an emotionally loaded keyword, as evidenced by the discrepancy in usage between “stress” and its Arabic equivalent “ضغط.” If “stress” were perceived similarly to “anxiety” and its Arabic equivalents, one would expect both “stress” and “ضغط” to exhibit similar search profiles. However, the data indicate otherwise, indicating that Kuwaitis may not use the keyword “stress” in the same manner as they do for “anxiety” and its Arabic equivalents. This discrepancy underscores the importance of considering cultural nuances and linguistic differences when interpreting online search behaviors related to mental health issues.

The contrast between the high search volume in English and the findings in Arabic can be attributed to linguistic nuances and the multiple meanings associated with the keyword “stress” in Arabic. In Arabic, “ضغط” (translation: “stress”; Arabizi “daght”) carries two distinct meanings. First, it can refer to mental stress, encompassing the body’s response to pressure triggered by various life matters, such as employment issues and relationship complications, which aligns with the intended meaning of the search listening project.

Secondly, “ضغط” can also denote “high blood pressure,” a prevalent health condition affecting many individuals in the region. Stress is a contributing factor to high blood pressure, heart attack, and stroke. Consequently, individuals, particularly the younger generation, may search for information related to both meanings. They may seek ways to manage high blood pressure, understand its causes, or explore strategies to cope with mental stress.

The higher search volume in Arabic could be attributed to individuals searching for information related to both meanings of “ضغط,” contributing to the overall volume. Without further context or analysis, it is challenging to ascertain the intended meaning of each search instance, highlighting the complexity of interpreting search data in multilingual contexts.

#### Anxiety, القلق and التوتر

The differences in search volume between the English term “anxiety” and its Arabic equivalents, “القلق” and “التوتر,” can be attributed to several factors. Firstly, “anxiety” is a widely recognized term, even among individuals who do not speak English fluently, and it is commonly used in conversation. This widespread familiarity with English terms may lead to more searches being conducted in English.

Second, a significant portion of the population in Kuwait is bilingual, with many individuals native speakers of English. This bilingualism could result in a higher number of searches being conducted in English than in Arabic. While bilingual individuals may conduct searches in both languages, native English speakers are likely to exclusively use English for their searches.

Additionally, the fact that there is only one term for “anxiety” in English, as opposed to two in Arabic, may concentrate the search volume in English. In Arabic, the search volume may be distributed between the two terms, “القلق” and “التوتر,” leading to a lower overall volume for each individual term. These factors collectively contribute to the larger search volume observed for “anxiety” in English compared to its Arabic equivalents.

The observed patterns in search volume for terms related to anxiety, despite occurring at different volumes, exhibited similar trends. Notably, there was a peak during the early morning hours, typically around 2–3 am, which aligns with the well-known phenomenon of anxiety attacks occurring more frequently during these hours. This peak is followed by a decline during typical Kuwaiti working hours when individuals are occupied with their tasks and responsibilities, diverting their attention away from anxiety triggers.

As the day progressed, particularly during the evening hours from 7 pm onwards, the search volume for anxiety-related terms began to rise again. This increase could be attributed to the time designated for rest and socializing in Arab culture, which provides individuals with opportunities to reflect on their feelings and search for information or support related to anxiety.

The subsequent rise in search volume until the early hours of the morning may be influenced by both cultural sleeping patterns and the impact of anxiety and stress. In Arab culture, late night hours are often associated with relaxation and contemplation, creating an environment conducive to introspection and online searches for information on anxiety. Overall, the observed patterns in search volume for anxiety-related terms reflect the complex interplay between cultural practices, daily routines, and mental health factors such as anxiety and stress.

#### Divorce and طلاق translation (divorce; Arabizi, altala8)

Data for this keyword depicted no discernible peaks in the Relative Search Volume (RSV) for the keyword “divorce.” The range between the highest and lowest search volumes was minimal, with the highest and lowest values at 28 and 23%, respectively. Conversely, when examining the RSV for the Arabic translation of “divorce,” “طلاق” (Arabizi, altala8), the RSV was higher, reaching a peak of 37% and a low of 17%.

A notable observation is the sharp decline in search volume between 2:30 am and 6:30 am, with some noise in the data recorded between 5:30 am and 6:30 am. This could be attributed to individuals preparing for work, commuting, or attending morning routines, leading to a decrease in online activities during these hours. The discrepancy in search volume between English and Arabic terms for “divorce” underscores the importance of linguistic and cultural considerations when analyzing search data.

The term “divorce” carries a significant emotional weight and parallels the emotional profile of anxiety. In Kuwait, where law enforcement operates primarily in Arabic, individuals seeking information on specific laws or details related to divorce cases are prompted to search for terms in Arabic. This preference for Arabic searches is influenced by Kuwait’s legal framework, which is exclusively conducted in Arabic. As a result, individuals navigating legal matters or seeking information on divorce-related issues are more likely to conduct searches using Arabic terms for “divorce.” This linguistic and legal context explains why searches for “divorce” are predominantly conducted in Arabic, highlighting the influence of language and legal systems on search behavior.

The Relative Search Volume (RSV) for the keyword “انفصال” (translation: separation; Arabizi, N.A) revealed a peak of 22% and a low of 9%. Interestingly, the highest RSV occurred between 5 am and 6:30 am, mirroring the pattern observed for searches related to the Arabic term for “divorce.” The relatively low search volume for the keyword “انفصال” can be attributed to cultural factors and societal norms prevalent in Kuwait. In Kuwaiti culture, separation is not commonly viewed as a viable solution for marital issues. Instead, couples facing difficulties in their relationship tend to either reconcile or pursue divorce if they believe that their future together is untenable.

Separation is perceived as uncommon and culturally unsupported in Arabic society. Even couples who are effectively separated often continue to live together while leading separate lives. Consequently, individuals may be less inclined to search for information or resources related to separation, contributing to the low RSV observed for this keyword. The cultural preference for either maintaining the marriage or pursuing divorce, coupled with societal norms regarding separation, likely underlie the limited search activity associated with the keyword “انفصال” in Kuwait.

## Conclusion

In conclusion, this study provides significant insights into the complex interplay between external factors, government responses, economic conditions, and the online manifestation of psychological distress. Through an examination of these dynamics on both global and local scales, this study adds depth to our understanding of how individuals manage and articulate their well-being within the digital landscape.

These findings highlight the importance of ongoing research efforts to further unravel the intricacies of this relationship and its impact on public health and policymaking. By delving deeper into these complexities, future studies can provide more effective interventions and policies aimed at supporting mental health and well-being in the digital age.

## Limitations and future research

Automated mining of large datasets offers significant advantages, including representativeness and the ability to analyse data on a broad scale. However, it is important to note that certain details, such as stratification by sex and age groups, may be overlooked in the broader analysis. Google Trends does not provide detailed insights into the exact reasons behind users’ searches for mental health-related terms examined in this study. For example, people may have been searching for information about their symptoms or looking for guidance on coping with mental health challenges during the COVID-19 pandemic. Despite this, engaging with big data brings fundamental benefits, such as increased efficiency, improved pricing strategies, and enhanced competition with larger enterprises.

Furthermore, leveraging big data enables businesses to focus on local preferences, which leads to increased sales and customer loyalty. Since individuals often own or independently collect these data, the cost of acquisition is minimal compared to traditional marketing methods, ensuring swift completion of tasks and cost-effectiveness.

While current efforts may not fully capture the nuances of search patterns across different demographics, further identification endeavors could provide deeper insights into the needs and preferences of various age groups and sexes. This knowledge would empower researchers to tailor programmatic advertisements more precisely, deliver targeted assistance, and enhance their overall effectiveness.

## Data Availability

The dataset is not fully disclosed due to commercial confidentiality. Requests to access these datasets should be directed to Ahmad Alsaber College of Business and Economics, American University of Kuwait (AUK), E-mail: aalsaber@auk.edu.kw.

## Footnotes

^1^https://www.bsg.ox.ac.uk/research/research-projects/covid-19-government-response-tracker

## References

[ref1] Abdel-Khalek A. M. Korayem A. S. (2018). The relationship between happiness, income, and unemployment rate in Arab and Western countries. Mank. Q. 59, 242–254. doi: 10.46469/mq.2018.59.2.6

[ref2] Ahmed S. Kia-Keating M. Tsai K. (2011). A structural model of racial discrimination, acculturative stress, and cultural resources among Arab american adolescents. Am. J. Community Psychol. 48, 181–192. doi: 10.1007/s10464-011-9424-3, PMID: 21287262

[ref3] Al Reshaid F. Tosun P. Yanar Gürce M. (2024). Cryptocurrencies as a means of payment in online shopping. Digital Policy Regulat. Govern. 26, 375–393. doi: 10.1108/DPRG-12-2023-0185

[ref4] AlBuloushi N. Al-Enzi A. AlReshaid F. Park K. M. Alsaber A. R. (2024). The powerful influence of connections: exploring the effects of Wasta informal networks on human resource development in Kuwait. Hum. Resour. Dev. Int. 1–30, 1–30. doi: 10.1080/13678868.2024.2378567

[ref9001] Al-Enzi A. Almutawaa D. Enezi D. Allougman N. (2023). An analysis of the academic effectiveness of hybrid learning: The experiences of faculty and students in Kuwait. doi: 10.1108/JARHE-09-2022-0283, PMID: 33281237

[ref5] Al-Kandari Y. Crews D. E. (2014). Age, social support and health among older kuwaitis. Qual. Ageing Older Adults 15, 171–184. doi: 10.1108/qaoa-10-2013-0031

[ref6] Al-Krenawi A. (2013). Mental health and polygamy: the Syrian case. World J. Psychiatry 3, 1–7. doi: 10.5498/wjp.v3.i1.1, PMID: 24175180 PMC3782180

[ref7] Al-Rasheed M. Alkadir A. Shuqiran K. Al-Aqeel S. Jahrami H. BaHammam A. (2021). The impact of quarantine on sleep quality and psychological distress during the covid-19 pandemic. Nature Sci. Sleep 13, 1037–1048. doi: 10.2147/nss.s313373 34262375 PMC8273741

[ref8] Al-Rubaie R. (2023). Unjust adjustment: dispelling the myth of English superiority within Kuwaiti STEM studies. Engl. Lang. Teach. 16:30. doi: 10.5539/elt.v16n4p30

[ref10] Bell V. Bishop D. V. Przybylski A. K. (2015). The debate over digital technology and young people. BMJ 351:h3064. doi: 10.1136/bmj.h3064 26268481

[ref11] Binder M. Coad A. (2012). Life satisfaction and self-employment: a matching approach. Small Bus. Econ. 40, 1009–1033. doi: 10.1007/s11187-011-9413-9

[ref12] Borges K. Figueiredo F. Souto R. (2017). Night eating syndrome and emotional states in university students. J. Human Growth Dev. 27:332. doi: 10.7322/jhgd.141277

[ref13] Boyce C. J. Wood A. M. Brown G. D. A. (2010). The dark side of conscientiousness: conscientious people experience greater drops in life satisfaction following unemployment. J. Res. Pers. 44, 535–539. doi: 10.1016/j.jrp.2010.05.001

[ref14] Brodeur A. Clark A. E. Fleche S. Powdthavee N. (2021). COVID-19, lockdowns and well-being: Evidence from Google trends. J. Public Econ. 193:104346. doi: 10.1016/j.jpubeco.2020.104346, PMID: 33281237 PMC7703221

[ref15] Cousin L. Beo V. Marcellin F. Torrente O. Mahé V. Valderas J.. (2023). Negative representations of night-shift work and mental health of public hospital healthcare workers in the covid-19 era (aladdin survey). BMC Health Serv. Res. 23:9101. doi: 10.1186/s12913-023-09101-7, PMID: 36814276 PMC9946706

[ref9002] Diotaiuti P. Valente G. Corrado S. Mancone S. (2023). Assessing decentering capacity in athletes: a moderated mediation model. Inter. J. Environ. Res. Public Health. 20:3324. doi: 10.3390/ijerph20043324 PMC9962655 36834019

[ref17] Dashti A. (2015). The role and status of the English language in Kuwait. English Today 31, 28–33. doi: 10.1017/S026607841500022X

[ref18] De’ R. Pandey N. Pal A. (2020). Impact of digital surge during Covid-19 pandemic: a viewpoint on research and practice. Int. J. Inf. Manag. 55:102171. doi: 10.1016/j.ijinfomgt.2020.102171 PMC7280123 32836633

[ref19] Diab-Bahman R. Al-Enzi A. (2020). The impact of the Covid-19 pandemic on conventional work settings: a local survey to investigate the current conditions of employees working remotely in Kuwait. Int. J. Sociol. Soc. Policy 40, 909–927. doi: 10.1108/IJSSP-07-2020-0262

[ref20] Diab-Bahman R. Al-Enzi A. (2021). The emotional impact of COVID-19 on undergrads. J. Clin. Schizophrenia Related Psychoses 15, 1–8. doi: 10.3371/CSRP.RBAE.170521

[ref21] Diener E. Wirtz D. Tov W. Kim-Prieto C. Choi D.-W. Oishi S.. (2010). New well-being measures: short scales to assess flourishing and positive and negative feelings. Soc. Indic. Res. 97, 143–156. doi: 10.1007/s11205-009-9493-y

[ref22] Eshel Y. Kimhi S. Marciano H. Adini B. (2022). Perceived partial social integration, levels of distress and resilience, and covid-19 vaccine rejection of jewish and Arab citizens of Israel. Front. Public Health 10:1021015. doi: 10.3389/fpubh.2022.1021015 36483251 PMC9723345

[ref23] Fino E. Fino V. Bonfrate I. Russo P. Mazzetti M. (2021). Helping patients connect remotely with their loved ones modulates distress in healthcare workers: tend-and-befriend hypothesis for covid-19 front liners. Eur. J. Psychotraumatol. 12:1968141. doi: 10.1080/20008198.2021.1968141, PMID: 34659653 PMC8519556

[ref24] Glowacz F. Schmits E. (2020). Psychological distress during the COVID-19 lockdown: the young adults most at risk. Psychiatry Res. 293:113486. doi: 10.1016/j.psychres.2020.113486, PMID: 33007682 PMC7518205

[ref25] Hale T. Angrist N. Goldszmidt R. Kira B. Petherick A. Phillips T.. (2021). A global panel database of pandemic policies (Oxford COVID-19 government response tracker). Nat. Hum. Behav. 5, 529–538. doi: 10.1038/s41562-021-01079-8, PMID: 33686204

[ref26] Hamdan A. (2009). Mental health needs of Arab women. Health Care Women Int. 30, 593–611. doi: 10.1080/07399330902928808 19492205

[ref27] Handique K. Teronpi J. (2022). A study on internet addiction disorder (IAD) and its related psychological factors during the COVID-19 lockdown. ECS Trans. 107, 16323–16341. doi: 10.1149/10701.16323ecst

[ref28] Hasan A. Tumah H. (2018). Determinants of quality of life among people diagnosed with schizophrenia at outpatient clinics. Perspect. Psychiatr. Care 55, 30–39. doi: 10.1111/ppc.12278 29645261

[ref29] Hassan G. Ventevogel P. Jefee-Bahloul H. Barkil-Oteo A. Kirmayer L. (2016). Mental health and psychosocial wellbeing of syrians affected by armed conflict. Epidemiol. Psychiatr. Sci. 25, 129–141. doi: 10.1017/s2045796016000044, PMID: 26829998 PMC6998596

[ref30] Hayes S. D. Anderson E. Carpenter B. W. (2022). Responsibility, stress and the well-being of school principals: how principals engaged in self-care during the COVID-19 crisis. J. Educ. Adm. 60, 403–418. doi: 10.1108/JEA-08-2021-0153

[ref31] Kornellia E. Syakurah R. A. (2023). Use of Google trends database during the COVID-19 pandemic: systematic review. Multidiscipl. Rev. 6:2023017. doi: 10.31893/multirev.2023017

[ref33] Lindheimer N. Karnouk C. Hahn E. Churbaji D. Schilz L. Rayes D.. (2020). Exploring the representation of depressive symptoms and the influence of stigma in arabic-speaking refugee outpatients. Front. Psych. 11:579057. doi: 10.3389/fpsyt.2020.579057, PMID: 33281643 PMC7689084

[ref34] Lindner C. Kotta I. Marschalko E. E. Szabo K. Kalcza-Janosi K. Retelsdorf J. (2022). Increased risk perception, distress intolerance and health anxiety in stricter lockdowns: self-control as a key protective factor in early response to the COVID-19 pandemic. Int. J. Environ. Res. Public Health 19:5098. doi: 10.3390/ijerph19095098, PMID: 35564492 PMC9100473

[ref35] Mostafa H. Alahmadi D. Jorob S. Murtada L. Aloufi R. Almeshaly S.. (2023). Psychological impact of online education on university students. Open access Macedonian. J. Med. Sci. 11, 9–14. doi: 10.3889/oamjms.2023.10917

[ref36] Orben A. Przybylski A. K. (2019). The association between adolescent well-being and digital technology use. Nat. Hum. Behav. 3, 173–182. doi: 10.1038/s41562-018-0506-1, PMID: 30944443

[ref37] Pereira D. Gross S. (2015). Social stressors at work, sleep, and recovery. Appl. Psychophysiol. Biofeedback 41, 93–101. doi: 10.1007/s10484-015-9317-6 26386866

[ref38] Ruotolo F. Ruggiero G. Cattaneo Z. Arioli M. Candini M. Frassinetti F.. (2023). Psychological reactions during and after a lockdown: self-efficacy as a protective factor of mental health. Int. J. Environ. Res. Public Health 20:6679. doi: 10.3390/ijerph20176679, PMID: 37681819 PMC10488210

[ref39] Saravanan C. Mahmoud I. Elshami W. Taha M. (2020). Knowledge, anxiety, fear, and psychological distress about covid-19 among university students in the United Arab Emirates. Front. Psych. 11:582189. doi: 10.3389/fpsyt.2020.582189, PMID: 33192728 PMC7642490

[ref40] Sharafeddine W. Diab-Bahman R. Al-Enzi A. (2024). The impact of fasting on workplace productivity and wellbeing: a review of fasting in Ramadan. Int. J. Product. Qual. Manag. 41, 32–48. doi: 10.1504/IJPQM.2024.136191

[ref41] Søvold L. E. Naslund J. A. Kousoulis A. A. Saxena S. Qoronfleh M. W. Grobler C.. (2021). Prioritizing the mental health and well-being of healthcare workers: an urgent global public health priority. Front. Public Health 9:679397. doi: 10.3389/fpubh.2021.679397, PMID: 34026720 PMC8137852

[ref42] Taheri M. Esmaeili A. Irandoust K. Mirmoezzi M. Souissi A. Laher I.. (2023a). Mental health, eating habits and physical activity levels of elite Iranian athletes during the COVID-19 pandemic. Sci. Sports 38, 527–533. doi: 10.1016/j.scispo.2023.01.002, PMID: 37362084 PMC10243596

[ref43] Taheri M. Irandoust K. Reynoso-Sánchez L. F. Muñoz-Helú H. Cruz-Morales K. N. Torres-Ramírez R.. (2023b). Effects of home confinement on physical activity, nutrition, and sleep quality during the COVID-19 outbreak in amateur and elite athletes. Front. Nutr. 10:1143340. doi: 10.3389/fnut.2023.1143340 37139442 PMC10150803

[ref44] Taylor A. Wright H. Lack L. (2008). Sleeping-in on the weekend delays circadian phase and increases sleepiness the following week. Sleep Biol. Rhythms 6, 172–179. doi: 10.1111/j.1479-8425.2008.00356.x

[ref45] Vahle-Hinz T. Bamberg E. Dettmers J. Friedrich N. Keller M. (2014). Effects of work stress on work-related rumination, restful sleep, and nocturnal heart rate variability experienced on workdays and weekends. J. Occup. Health Psychol. 19, 217–230. doi: 10.1037/a0036009, PMID: 24635734

[ref46] Vaidyanathan U. Sun Y. Shekel T. Chou K. Galea S. Gabrilovich E.. (2022). An evaluation of internet searches as a marker of trends in population mental health in the US. Sci. Rep. 12:8946. doi: 10.1038/s41598-022-12952-5, PMID: 35624317 PMC9136741

[ref47] Xu X. Liu J. Liu J. (2023). The effect of social media environments on online emotional disclosure: tie strength, network size and self-reference. Online Inf. Rev. 48, 390–408. doi: 10.1108/oir-04-2022-0245

